# Economic impact of bovine cysticercosis and taeniosis caused by *Taenia saginata* in Belgium

**DOI:** 10.1186/s13071-018-2804-x

**Published:** 2018-04-13

**Authors:** Famke Jansen, Pierre Dorny, Chiara Trevisan, Veronique Dermauw, Minerva Laranjo-González, Alberto Allepuz, Céline Dupuy, Meryam Krit, Sarah Gabriël, Brecht Devleesschauwer

**Affiliations:** 1 Department of Biomedical Sciences, Institute of Tropical Medicine, 155 Nationalestraat, B-2000 Antwerp, Belgium; 2 Laboratory of Parasitology, Faculty of Veterinary Medicine, Ghent University, 133 Salisburylaan, B-9820 Merelbeke, Belgium; 3 IRTA, Centre de Recerca en Sanitat Animal (CReSA, IRTA-UAB), Campus de la Universitat Autònoma de Barcelona, Bellaterra, 08193 Barcelona, Spain; 4 Departament de Sanitat i Anatomia Animals, Universitat Autònoma de Barcelona (UAB), 08193 Bellaterra, Barcelona, Spain; 5 General directorate for food, Slaughterhouses and cutting plants board, 69401 Lyon, France; 6 Department of Veterinary Public Health and Food Safety, Faculty of Veterinary Medicine, Ghent University, 133 Salisburylaan, B-9820 Merelbeke, Belgium; 7 Department of Public Health and Surveillance, Scientific Institute of Public Health (WIV-ISP), Juliette Wytsmanstraat 14, B-1050 Brussels, Belgium

**Keywords:** *Taenia saginata*, Bovine cysticercosis, Economic impact

## Abstract

**Background:**

Bovine cysticercosis (BCC) (due to *Taenia saginata*) is often claimed to cause considerable economic losses to the livestock industry, particularly in beef cattle, but recent studies estimating the economic impact are lacking. The current study aimed to quantify the annual economic impact of BCC in Belgium from 2012 to 2016, by gathering data from diverse sources in the meat and human health sectors.

**Results:**

In Belgium, on average, 15 carcasses with generalised infections and 1168 carcasses with localised ones are detected upon meat inspection each year. The highest proportion of the total economic losses due to bovine cysticercosis were borne by the cattle owners with an average economic cost of €3,408,455/year: €2,954,061/year due to BCC insurance, €453,024/year due to value losses of beef of uninsured carcasses (i.e. freezing process) and €1370/year due to destruction costs of uninsured carcasses with generalised infections. The slaughterhouses suffered an economic impact of €210,806/year. They were responsible for inspection costs related to meat inspection in general, administration, processing and deboning of infected carcasses (€597,856/year), value losses (€34,848/year) and destruction costs (€105/year) of carcasses insured by the slaughterhouses (unofficial insurance) (5% of slaughtered animals). On the other hand, the slaughterhouses gained a total of €422,004/year due to unofficial insurance fees. Thirty percent of all slaughtered animals were officially insured against BCC and the insurance company generated an income of €2,322,337/year. The economic impact related to taeniosis (10,991 patients annually) amounted to a maximum of €795,858/year.

**Conclusion:**

BCC and taeniosis due to *T. saginata* have a large economic impact in Belgium, mainly due to the insurance costs for BCC. These results indicate the need for reducing the number of BCC and taeniosis cases to avoid the costs and losses related to this parasite.

## Background

*Taenia* spp. are cestodes occurring worldwide, both in low and high income countries. *Taenia solium*, the pork tapeworm, causes severe human health problems, while the other two species, *Taenia saginata* and *Taenia asiatica*, are of limited clinical importance but may inflict considerable economic losses on the livestock industry [1]. While *T. asiatica* has never been reported to occur in Europe and *T. solium* is no longer endemic, infections with *T. saginata* remain common [2]. The adult beef tapeworm resides in the intestinal lumen of the human final host (taeniosis), usually leading to only mild signs and symptoms such as abdominal discomfort, mild diarrhoea and weight loss. The most common symptom of the disease is anal pruritus caused by the actively migrating proglottids [2, 3]. People become infected by consuming raw or undercooked beef containing viable cysticerci, the metacestode larval stage found primarily in the muscles of the bovine intermediate host (bovine cysticercosis, BCC) [4]. In the European Union, diagnosis of BCC in the slaughterhouses is based on official meat inspection (MI) consisting of visual inspection of the oesophagus, tongue, diaphragm and visible muscle surfaces and incisions made in the heart and masseter muscles (EC Regulation 854/2004). The estimated prevalence of BCC in Belgium, based on MI, was reported to be 0.23% [5–9], yet official figures for prevalence tend to be underestimations [10–13] as MI is known to have a low sensitivity.

It is generally assumed that BCC is responsible for important economic losses in the meat sector, particularly for beef cattle [1, 14, 15]. EC regulations dictate that carcasses diagnosed with generalised/heavy BCC (i.e. multiple cysticerci found during MI) are condemned and destroyed, while those with localised/light infections (only one or a few cysticerci found during MI) must be frozen (at least 10 days at -18 °C) prior to consumption (EC Regulation 854/2004). Freezing of beef substantially decreases the value (30–45%) [16]. Overall, however, there is a lack of recent studies estimating the economic impact of BCC and taeniosis in Europe [3]. A review by Murrell [17] estimated the value loss for a carcass due to bovine cysticercosis at US $234/carcass and medical costs for taeniosis at US$ 111/patient in industrialised countries in 1990. In England, costs due to BCC were estimated at GB £100/carcass or GB £4.0 million annually in 1999 [18]. In Belgium, the economic impact was estimated at €600,000 (24,262,400 Belgian franc) in 1987, including consultations and medication costs for patients (€12.5/patient) and value losses for bovine carcasses (€620/carcass) [16]. The current study aimed to provide an updated estimate of the annual economic impact caused by *T. saginata* in Belgium for the meat and the human health sectors, based on recent data (2012–2016).

## Methods

### Study site/population

The economic impact due to *T. saginata* was estimated for the reference period 2012–2016 in Belgium, an industrialised country with an estimated 11,348,160 inhabitants in 2016 [19]. An average of 511,528 adult cattle and 340,221 calves are slaughtered in the country on a yearly basis [5–9].

### Collection of information from the animal/meat sector and official veterinary services

Official information regarding the average number of carcasses with generalised (15 animals) and localised [1168.2 animals (abbreviated for calculations to 1168)] cysticercosis per year, based on routine meat inspection, was collected from the Annual reports of the Federal Agency for the Safety of the Food Chain (FASFC) [5–9]. For BCC positive carcasses, additional information was gathered (sex, birth date, slaughter date, slaughter weight and slaughterhouse) by personal communication with FASFC. All BCC positive carcasses were found to originate from adult cattle, therefore all further estimations were based on adult cattle data. The value (€/100 kg) of beef (per week) according to the SEUROP classification (carcass classification defined by the European Union) and subdivided into bulls, cows and heifers, was available on the website of the Department of Agriculture and Fisheries [20]. The SEUROP classification subdivides cattle carcasses into quality categories depending on the conformation of the carcass (S = superior; E = excellent; U = very good; R = good; O = average; and P = low) [21].

A single animal carcass destruction company is present in Belgium and was contacted for information on the destruction costs of carcasses with generalised infections. Burning of carcasses costed €187.67/ton and transport to the burning facility costed €50/hour.

Furthermore, Belgium has one official insurance company for BCC and information about the insurance policy and the cost of the insurance fee was provided by its owner. Approximately 30% of all bovines were insured through the official insurance company, prior to slaughter (mostly beef cattle). The insurance fee was between €8–25, depending on the number of animals insured by the same animal owner (administrative costs diminished with an increasing number of insured animals). Apart from BCC, the insurance also covered sarcosporidiosis, so the true cost for BCC insurance specifically was not known. In some slaughterhouses, it was also possible to obtain an unofficial insurance. Information concerning this system was gathered from the president of the Professional Association of Flemish Cattle dealers and Meat producers (VVV). The unofficial insurance fee was within the same price range as the official one and approximately 5% of animals were unofficially insured at the slaughterhouses (mostly beef cattle).

Information on (i) the inspection costs, (ii) the time inspectors at the slaughter houses need for proceedings related to BCC, as well as (iii) the value loss of beef due to freezing, was obtained from FASFC, the insurance company, the Belgian Meat Federation (FEBEV) and a questionnaire sent to all slaughterhouses in Belgium. Eight of 36 slaughterhouses replied and answers were formulated by meat inspectors or other contributors of the slaughterhouses. The time an inspector needs for the proceedings related to BCC can be divided into three components: (i) the time during meat inspection addressed specifically to cysticercosis; (ii) the time necessary for preparing the carcasses for shipment to the freezing, deboning or destruction facilities (including administration) (this is defined further as ‘processing’ and is done by the inspector himself); and (iii) the deboning process taking place before freezing of the carcasses with localised infections during which an inspector needs to be present. The information gathered from these sources was combined and validated through discussion with experts at the FASFC. The inspection fee, which slaughterhouses are obliged to pay to FASFC for the presence of meat inspectors during slaughtering, was rated at €75/hour. The time attributed specifically to the detection of cysticercosis was estimated between 15–60 seconds (s)/carcass. In case a cysticercosis positive carcass was detected (generalised or localised), the time needed to complete the administration involved and the supervision during the preparation for shipment to the deboning, freezing or destruction facilities (processing) was estimated at 30–90 minutes (min)/ carcass. Carcasses with localised infections were deboned under supervision of an inspector and parts were frozen separately. The deboning process took between 60–90 min/carcass. The value loss of a frozen carcass is currently estimated to range between 40–70%. This percentage includes the costs associated with the freezing process, the transport to the freezing facility and the weight loss of the carcass/beef due to freezing. Although the deboning process introduces an additional inspection cost, the process itself will reduce the total value loss, as a complete carcass needs a significant amount of time to thaw and needs to be processed immediately. When a carcass is deboned prior to freezing, thawing is faster and processing of the meat is much easier and more efficient. Carcasses with generalised infections are condemned and their total value is lost.

### Collection of information from the human health sector

Taeniosis is not a notifiable disease in Belgium. Therefore, prevalence was estimated from the sales numbers of taenicidal drugs [2]. In Belgium, the prescribed drug for taeniosis is niclosamide, produced and commercialized as ‘Yomesan®’. Over the last five years, on average 10,991 patients were treated per year (personal communication Bayer Pharma AG). The price for one dose of Yomesan® was €6.29 [22]. We assume all patients received treatment.

No information was gathered about the proportion of patients with a suspected tapeworm who consulted a physician. We assumed that patients consulting a physician would generally have a follow-up appointment ensuring the treatment was successful. In general, a single consultation had a cost of €25 for the patient, of which €21 was reimbursed by the health care sector (expert opinion). Patients with special reimbursement schemes were not accounted for in the estimations. Since Belgian phycisians rarely encounter a patient with a tapeworm (personal communication physicians), we chose for the worst-case scenario and assumed that all physicians filed for a diagnostic test. One test costs €16.12 and is paid by the patient (personal communication, Institute of Tropical Medicine Antwerp).

### Analyses

A model was written in the R programming language (R Core Team, 2014), combining the information described above, to calculate the economic burden of BCC, and more specifically the value losses suffered from infected carcasses, inspection costs, destruction costs for carcasses with generalized infections, insurance costs and costs related to human health (see below). The model performs the calculations for the economic impact as follows.

#### Estimation of value losses suffered from infected carcasses

**Fig. 1:**
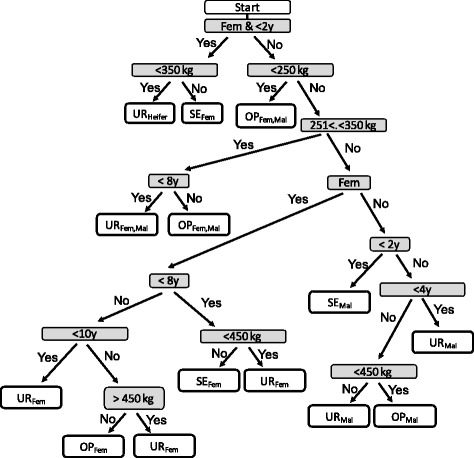
Flowchart for class determination of each individual animal. *Abbreviations*: SE, S and E class; UR, U and R class; OP, O and P class; Fem, female; Mal, male; y, years

The data for the cysticercosis positive carcasses with localised infections (5841 animals) obtained during the study period (personal communication FASFC) contained missing values. For six animals, the exact slaughter date was missing and only the year of slaughter was provided. We chose to replace the missing value with the first of July of the year of slaughter (mid-year). Missing weight data (20 animals) were replaced with the average weight calculated from all other positive carcasses. Animals with missing sex values (838) were excluded from further calculations. Therefore, information for a total of 5003 carcasses with localised infections was used for calculating the value loss. For carcasses with generalised infections (74 animals), the same substitutions were made for missing values of weight (one animal) and slaughter date (one animal). Expert opinion was used to determine the class (SE, UR or OP) of each cysticercosis positive carcass between 2012 and 2016 (Fig. 1). The code obtained for each animal was linked to the corresponding beef value to assign the corresponding value to each carcass [20]. Monthly, instead of weekly, average beef values were calculated and an average value was calculated for the S and E classes combined (SE), U and R classes combined (UR) and O and P classes combined (OP).

Expert opinion indicated that slaughter yield ranged between 55–70% for Belgian cattle [23, 24]. We chose an average slaughter yield of 62.5% for estimating the cold carcass weight from the slaughter weight. The total value of each carcass was calculated using the cold carcass weight and the assigned value for beef. For carcasses with generalised infections, the entire value of the carcass was lost. Carcasses with localised infections needed to undergo freezing and the value loss due to freezing depended on the value of the beef. Losses were lower for high value beef than for low value beef (e.g. dairy cattle) (personal communication FASFC, insurance company, FEBEV and slaughterhouses). To determine the value loss, we assigned the lowest percentage value loss (40%) to the most valuable carcass in the data and the highest percentage value loss (70%) to the least valuable carcass. All carcasses with values in between received percentage value losses respective to their estimated value by linear interpolation. The actual value loss was calculated by multiplying these percentages by the total carcass value. The individual value losses were summed, divided by the sample size and multiplied by the annual number of cysticercosis cases to calculate an average total value loss per year for Belgium.

#### Estimation of inspection costs

The average time, based on the estimated time limits, was used to determine the time spent on each carcass for searching for BCC during meat inspection (37.5 s/carcass), for processing the infected carcasses (60 min) and for deboning the localised infected carcasses (75 min) (personal communication FASFC, insurance company, FEBEV and slaughterhouses). Cost per carcass was estimated by multiplying these values by the inspection fee (€75/hour) (personal communication FASFC, insurance company, FEBEV and slaughterhouses). The annual costs for these processes were estimated by simply multiplying the previous estimates by the number of animals in the population (slaughtered cattle for ‘MI’, all infected carcasses for ‘processing infected carcass’ and carcasses with localised infections for ‘deboning’).

#### Estimation of destruction costs for carcasses with generalised infections

For the annual costs due to the destruction of generalised infected carcasses, their average annual weight over the study period (personal communication FASFC) was multiplied by the cost of destruction (€187.67/ton) (personal communication destruction company). To estimate costs of transport of infected carcasses, the average annual distance (km) was calculated based on the fastest route from the slaughterhouses in which the respective positive carcasses were found, to the destruction facility. This value was used to determine the duration of transport, based on an average speed of 60 km/h, and consequently the cost of transport. The route chosen was probably not the actual route taken by the driver since multiple pick-ups could have been combined in one drive and transportation costs were most likely divided amongst pick-up places. However, it remains the best estimate possible for the total cost for the transport to the destruction facility.

#### Estimation of insurance fee

An average annual cost was calculated for all officially and unofficially insured animals, using information about the proportion of animals that are officially (30%) and unofficially (5%) insured prior to slaughter and the average cost for the insurance (€16.5) (personal communication insurance company, VVV).

#### Estimation of costs related to human health

Given the lack of information on the proportion of patients consulting a physician with a suspected tapeworm infection, values between zero and one (using increments of 0.1) were used for this variable in the model and calculations were made for all possible values. The annual cost for consultations was estimated by multiplying the number of taeniosis cases (10,991; based on sale of ‘Yomesan®’) by the proportion of people consulting a physician, the number of consultations per patient (two) and the cost of a consultation for both patients (€4) and the health care system (€21).

The total cost for diagnostic tests was calculated by multiplying the number of taeniosis cases by the assumed proportion of patients that go to a physician, the proportion of physicians ordering diagnostic tests (one) and the cost of the diagnostic test (€16.12) (personal communication, Institute of Tropical Medicine). The cost for the drug was estimated by multiplying the number of taeniosis cases by the price of ‘Yomesan®’ (€6.29) [22], assuming all patients received treatment.

## Results

### Animal/meat sector

The average annual estimated costs for the meat sector can be found in Table 1. The annual economic impact for BCC for the reference period 2012–2016, estimated in this study, was interpreted with respect to the different parties involved: the animal owners, the slaughterhouses and the insurance company (Table 2). The insurance company (30% animals insured) and the slaughterhouses (5% animals insured) were responsible for the value losses and destruction costs of the insured animals. The same costs were borne by the animal owner for the uninsured animals.

**Table 1:** Summary of the estimated annual costs related to bovine cysticercosis in Belgium: value loss, costs related to inspection at the slaughterhouses, costs related to the destruction of carcasses with generalised infections and insurance costs

Category of cost	Type of cost	€/year	€/unit of interest
Value loss	Generalised infections	12,874	(€858/carcass generalised infection)
	Localised infections	684,087	(€586/carcass localised infection)
	Total value loss	696,961	
Inspection	Meat inspection	399,631	(€0.78/slaughtered animal)
	Processing infected carcass	88,725	(€75/infected carcass)
	Deboning carcasses with localised infection	109,500	(€93.75/carcass localised infection)
	Total inspection cost	597,856	
Destruction	Destruction cost	1023	(€68.2/carcass generalised infection)
	Transport to destruction facility	1084	(€72.3/carcass generalised infection)
	Total destruction cost	2107	(€140.5/carcass generalised infection)
Insurance	Officially insured animals	2,532,057	(€16.5/insured animal)
	Unofficially insured animals	422,004	(€16.5/insured animal)
	Total insurance cost	2,954,061	
Total		4,250,985

**Table 2:** Summary of the estimated annual losses (negative values) and income (positive values) related to bovine cysticercosis in Belgium for the animal owners, the slaughterhouses and the insurance company

Sector	Category of cost	€/year
Animal owner	Insurance	-2,954,061
	Value loss	-453,024
	Destruction	-1370
	Total	-3,408,455
Slaughterhouses	Insurance	422,004
	Value loss	-34,848
	Destruction	-105
	Inspection	-597,856
	Total	-210,806
Insurance company	Insurance	2,532,057
	Value loss	-209,088
	Destruction	-632
	Total	2,322,337

### Human health sector

**Fig. 2:**
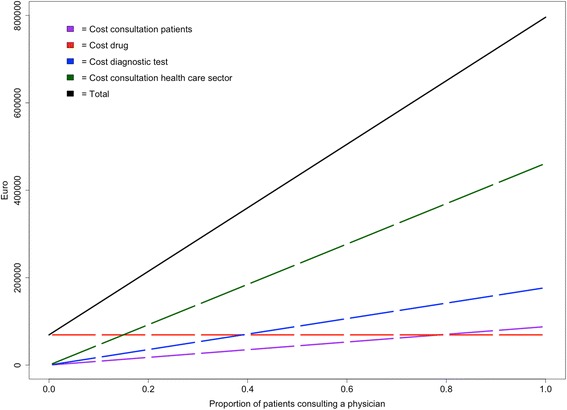
Costs related to consultations for patients and for the human health care system, costs for drugs, diagnostic tests and the total costs, relative to the proportion of patients consulting a physician

Figure 2 shows the costs for the human health sector, for each value assigned to the proportion of patients consulting a physician between zero and one. Annual drug costs were independent of the proportion of people consulting a physician and amounted to €69,133 (Fig. 2, red line). If all patients with taeniosis were to consult a physician, the consultation costs would amount to a maximum of €87,928 and €461,622 for patients (Fig. 2, purple line) and for the human health care system (Fig. 2, green line), respectively, and to a maximum of €177,175 for diagnostic tests (Fig. 2, blue line). In total, this means that the annual cost for the human health care sector varies from €69,133 (€6.29/patient) in case no patients consult a physician, to €795,858 (€72.4/patient) if all patients consult a physician, all physicians request a diagnostic test and all patients consult the physician again for confirmation of successful treatment (Fig. 2, black line).

## Discussion

We quantified the economic impact of *T. saginata* in Belgium in a comprehensive way for both the animal/meat sector and the human health sector. The highest economic cost was borne by the animal owners, with a total annual cost of €3,408,455, mostly due to insurance fees. Slaughterhouses suffer a substantially smaller loss of €210,806/year. This number is an average value for all Belgian slaughterhouses combined. However, not all slaughterhouses offer the unofficial insurance for BCC to cattle owners, so they will not generate an income to compensate for losses. Some slaughterhouses will cooperate directly with the official insurance company and insure all animals entering the slaughterhouse. The balance between loss and income thus varies between slaughterhouses.

The value loss per carcass was estimated for industrial countries in previous studies: €620 (25,000 Belgian franc) per carcass for Belgian cattle (in 1987) [16], €198 (US$ 234) per carcass for cattle in industrialised countries (in 1990) [17] and €113.6 (GB£ 100) per carcass in the United Kingdom (1999) [18]. Our current estimate (€586/carcass) is higher than the estimates made for industrialised countries and the UK, but lower than the estimate for Belgium originating from 1987. This previous estimate was a rather rough estimate based on the average value of all slaughtered cattle, and not considering solely the BCC positive animals (as was done in the current study). In Belgium, high value carcasses (e.g. Belgian Blue breed) are easily worth €2500, whereas the average annual value of an infected carcass was estimated to be €1073 in the current study period. The lower average value loss found in the current study indicates that BCC infections are mainly found in low value carcasses. In the Belgian livestock industry, low value carcasses are mostly culled dairy cows. The occurrence of BCC has been positively correlated with increasing age and being female [11, 25–28].

The percentage value loss per carcass was always considered to be between 30–45% [16], yet has now increased to between 40–70% (personal communication FASFC, insurance company, FEBEV and slaughterhouses). This is most likely due to an increase in the costs related to the freezing process. Comparison with the UK and industrialised countries estimates is more realistic and shows an important increase in value loss per carcass during the last 20–30 years.

Our study had some limitations. While collecting data, extensive information for some parameters could not be obtained, so distributions could not be included in the model for these parameters. Slaughter yield is between 55–70% for Belgian cattle (with exceptions), depending on the breed/physique of the animal (dairy/beef), the stomach content, the conformation, the fatness, the bone structure, the feed received during life, age, weight, the methods used for deboning and the slaughter weight. Many of these factors were unknown for the infected carcasses so we chose an average slaughter yield of 62.5% for our estimations of value loss [23, 24]. The time spent searching for BCC during meat inspection and the time for processing and for deboning of infected carcasses was obtained from various sources (personal communication FASFC, insurance company, FEBEV and slaughterhouses), allowing us to identify a range of possible values. There were no indications as to what time value might be the most probable. The same is true for the cost of the insurance for each animal, which depends on many factors that were not specified per animal by the insurance company. Therefore, the average value was used for these parameters.

Inconsistencies were uncovered between the official number of cysticercosis cases and the number of cysticercosis cases provided by the owner of the insurance company [e.g. amongst the insured animals (30% of all slaughtered animals), 21 animals with generalised infections were reported in 2013, while there were only 16 according to official records in the total population]. Other common differential diagnoses such as abscesses, *Sarcocystis* infections or granulomas [29] could lead to misdiagnosis and misclassification of some animals during the administrative process, making it difficult to retrieve correct information about cysticercosis. This might indicate that the numbers of the insurance company were an overestimation of the total BCC numbers or that the official numbers were an underestimation. If the latter is true, estimations made for the economic impact in this study were also an underestimation. When extrapolating the numbers received by the insurance company to annual averages, there would be 63.5 carcasses with generalised infections (based on data from 2013 until the end of 2016) and 1406 carcasses with localised infections (based on data from 2016) per year. With these higher numbers of infected carcasses, the annual cost for the animal owner would increase to €3,530,547 (compared to €3,408,455), the costs for the slaughterhouses would increase to €263,996 (compared to €210,806) and the insurance company would have an income of €2,282,337 (compared to €2,322,337). These higher estimates did not reveal a substantial increase in the economic impact. Eventually, irrespective of the scenario, the largest proportion of the economic losses suffered will be borne by the cattle owners.

As previously mentioned, the official insurance policy includes an insurance against sarcosporidiosis and for the unofficial insurance, it is not clear which conditions are being covered by the policy. Therefore, it is not clear which proportion of the insurance fee is attributed to cysticercosis. However, the number of sarcosporidiosis cases (currently 85 cases/year, personal communication FASFC) is much lower than the number of cysticercosis cases so the estimate based on the entire insurance fee remains the best possible estimate, even though it is an overestimation.

The costs related to the human health sector constitute a much smaller portion of the total economic impact compared to the animal/meat sector, yet imply an maximum annual cost of €795,858, including consultations, diagnostic tests and medication. For these estimates, the assumption was made that all patients will have a follow up consultation and all physicians will demand a diagnostic test for each patient. These assumptions might be overestimations compared to the real situation but it was not within the limits of this study to gather exact data for these parameters.

The drug used for taeniosis, Yomesan®, is not 100% effective [30], so some patients might need an extra dose to expel the tapeworm. Yomesan® can also be used against other tapeworm infections such as *T. solium* and *Diphyllobothrium latum*, but those infections are non-endemic and very rarely diagnosed in Belgium ([31, 32] Potters, I., Institute of Tropical Medicine, personal communication). Praziquantel, commonly used in neighbouring countries for treatment of taeniosis, is not available in Belgium in regular pharmacies and is therefore not prescribed for taeniosis. Mebendazole (‘Vermox®’) is available without prescription in regular pharmacies and may erroneously be sold by pharmacists to taeniosis patients when they suspect a nematode infection (mostly *Enterobius vermicularis*). However, the recommended dose of Mebendazole for curing taeniosis is three times higher than for *E. vermicularis*. A single Mebendazole dose is likely to be inefficient for taeniosis and patients will require another treatment. It is not known how many patients received Mebendazole for treatment of taeniosis. Due to this lack of knowledge and since the drug sales numbers of Yomesan® are generally used to estimate the annual number of taeniosis patients [33], this method was considered the best option.

The cost per taeniosis patient was estimated in 1987 to be €12.5 in Belgium (including consultations and medication) [16]. This cost has increased to €56.3 in the current study (only including consultations and medication for comparison). The estimate made by Murrel [17] for industrialised countries of US$ 111/patient also included the possible wage loss a patient might experience due to a tapeworm, by consulting a physician or hospital or for the time they are unfit for work (€93.8/patient). This was not estimated in the current study.

Several factors listed below can be considered as a cost related to BCC or taeniosis but were not included in the calculations since they were either rare, case-depending or implied a very small proportion of the total cost. However, they would even further increase the economic impact of the parasite on the animal and human health sector.

- The animal owner of a positive carcass can make the personal decision to take several actions that can lead to additional costs: official or private veterinarians could perform further investigations on the remaining animals on the farm, attempt to trace the infection or give advice on how to prevent further infections, farm workers could be tested for *T. saginata* infection and informed on how to handle this infection, and new measures for the prevention of infections could be implemented (e.g. water filters).
- Veterinarians performing the meat inspection at the slaughterhouses regularly attend training sessions and continue professional education, and slaughterhouses are frequently audited.
- Research projects exploring all aspects of the parasite/disease need funding.
- Examples exist of non-specific complications associated with *T. saginata* infection in humans (e.g. cholangitis, peritonitis, post-appendectomy fecal fistula) [34–36], including a possible need for a hospitalization. In Belgium, this is extremely rare. According to official data, in 2010 and 2014 none and in 2008, 2009, 2011, 2012 and 2013 less than five patients were hospitalised with *T. saginata* as a primary diagnosis and without indication of severity. No data were available for 2015 and 2016. These data are not always correct and the distinction between *T. saginata* and other *Taenia* spp. is not always made [31]. Psychological stress of patients due to the presence of a tapeworm could cause additional costs not included in the analysis.
- In many studies on economic burden, transportation costs to and from the physician and pharmacy are included. In Belgium, medical practices and pharmacies are present in every village, making these costs negligible.
- Sewage is treated before wastewater enters the waterways. Sadly, current wastewater management not only fails to halt, but rather contributes to the dissemination of eggs in the environment, leading to contaminated waterbodies and surface water [2]. Sewage sludge is no longer used in agriculture to fertilize pastures in Belgium, but in other European countries it is still allowed and can pose a higher risk for cattle [37].
- For many diseases, part of calculating the burden of disease is to quantify health losses using Disability-adjusted life years (DALYs). One DALY is defined as one lost year of healthy life. For taeniosis caused by *T. saginata*, the DALY is probably very low, since most infections remain asymptomatic. For those few patients that will experience mild abdominal pain, DALYs can be estimated to be 0.012/year, which is still very low [38].

Meat inspection is known to have a low sensitivity and a recent study in Belgium found a much higher prevalence (36%) of BCC than previously thought, using a combination of several detection techniques (MI, dissection of the predilection sites, B158/B60 Ag-ELISA and excretory/secretory Ab-ELISA) [13]. Undetected viable cysticerci can enter the food chain and potentially cause taeniosis in humans when they eat raw or undercooked beef. Introducing a new and more sensitive detection technique at slaughter might be one way of reducing the number of cysticerci in the food chain. This would imply that more carcasses are condemned or need to be frozen after slaughter, introducing a higher cost at first, but leading to a reduction in the number of taeniosis patients, subsequently in the number of cattle with cysticerci and eventually in the total economic impact due to BCC and taeniosis. A model assessing new detection techniques and their impact on prevalence of cysticercosis and taeniosis and the economic impact in Belgium is currently being developed by the authors of this study.

Human tapeworm carriers are essential for maintenaining transmission, therefore, the responsibility of the medical sector and general population in the control of this parasite should not be neglected. People should be made aware of the risk they pose towards cattle when eating raw or undercooked beef and developing a tapeworm. There is an urgent need within the medical sector for information campaigns for physicians and patients on how to treat patients and how to correctly dispose of the expelled tapeworm, in order to reduce the infection risk for cattle and the economic impact on cattle owners.

## Conclusions

In conclusion, BCC and taeniosis due to *T. saginata* cause a relatively large economic impact in Belgium, especially due to the insurance costs for BCC, borne by the farmers. These results demonstrate the need to reduce the number of BCC and taeniosis cases to, in turn, avoid or reduce the costs and losses related to this parasite for the animal and human health sectors.

## Abbreviations

BCC: bovine cysticercosis
EC: European Commission
MI: meat inspection
FASFC: Federal Agency for Safety of the Food Chain
DALY: disability-adjusted life years
